# PROMOTING DIGITAL HEALTH EQUITY THROUGH COLLABORATIVE PARTNERSHIPS WITH CHINESE AMERICAN COMMUNITIES

**DOI:** 10.1093/geroni/igae098.0698

**Published:** 2024-12-31

**Authors:** Jingyi Li, Jinchen Xie, Yanjing Liang, Jingyi Dong, Weichao Yuwen

**Affiliations:** University of Washington Tacoma, Tacoma, Washington, United States; University of Washington, Seattle, Washington, United States; University of Washington, Seattle, Washington, United States; Seattle Pacific University, Seattle, Washington, United States; University of Washington Tacoma, Tacoma, Washington, United States

## Abstract

Despite overwhelming and disparate needs, the use of supportive healthcare programs is lower among Chinese American caregivers, in large part due to the lack of accessible and culturally and linguistically appropriate care. Digital health tools have the potential to support caregivers’ needs. In collaboration with community partners, we conducted a qualitative study to identify opportunities using digital health tools to address caregiving needs among families caring for Chinese American older adults. Four focus groups with 21 staff from four local community-based organizations serving Chinese American families were conducted to explore staff’s experience supporting Chinese American families and discuss their perspectives on opportunities to use digital health tools to support caregiving needs. Inductive thematic analysis revealed that digital health tools have the potential to support Chinese American families caring for older adults by (1) offering real-time and trustworthy informational support on caregiving, (2) guiding caregivers through system navigation and service discovery, (3) sharing caregiving stories for scenario-based learning, (4) suggesting communication strategies for adult children caregivers, and (5) enabling family check-ins before or between case manager consultations. Additionally, staff raised concerns regarding digital health tools’ capacity to offer culturally sensitive emotional support, alongside skepticism about its potential to substitute valuable human social interactions. The findings from this study lay the foundation for future research on culturally adapting digital health tools for promoting the mental health and well-being of Chinese American family caregivers.

